# Comparative biological effects of spherical noble metal nanoparticles (Rh, Pd, Ag, Pt, Au) with 4–8 nm diameter

**DOI:** 10.3762/bjnano.9.258

**Published:** 2018-10-29

**Authors:** Alexander Rostek, Marina Breisch, Kevin Pappert, Kateryna Loza, Marc Heggen, Manfred Köller, Christina Sengstock, Matthias Epple

**Affiliations:** 1 Inorganic Chemistry and Center for Nanointegration Duisburg-Essen (CeNIDE), University of Duisburg-Essen, Universitaetsstr. 5–7, D-45117 Essen, Germany. Fax: +49 201 1832621; Tel: +49 201 1832402; E-mail: matthias.epple@uni-due.de; 2 Bergmannsheil University Hospital/Surgical Research, Ruhr-University of Bochum, Bürkle-de-la-Camp-Platz 1, 44789 Bochum, Germany; 3 Ernst Ruska-Center and Peter Gruenberg Institute, Forschungszentrum Juelich GmbH, 52425 Juelich, Germany

**Keywords:** cytotoxicity, electron microscopy, metals, nanoparticles, nanotoxicity

## Abstract

For a comparative cytotoxicity study, nanoparticles of the noble metals Rh, Pd, Ag, Pt, and Au (spherical, average diameter 4 to 8 nm) were prepared by reduction in water and colloidally stabilized with poly(*N*-vinyl pyrrolidone) (PVP). Thus, their shape, size, and surface functionalization were all the same. Size and morphology of the nanoparticles were determined by dynamic light scattering (DLS), analytical disc centrifugation (differential centrifugal sedimentation, DCS), and high-resolution transmission electron microscopy (HRTEM). Cell-biological experiments were performed to determine the effect of particle exposure on the viability of human mesenchymal stem cells (hMSCs). Except for silver, no adverse effect of any of the metal nanoparticles was observed for concentrations up to 50 ppm (50 mg L^−1^) incubated for 24 h, indicating that noble metal nanoparticles (rhodium, palladium, platinum, gold) that do not release ions are not cytotoxic under these conditions.

## Introduction

Inorganic and metallic nanoparticles represent a well-established part of materials science, heterogeneous catalysis, and nanomedicine [1–3]. Noble metal nanoparticles are of particular importance due to their diverse properties such as surface plasmon resonance, chemical inertness, and antibacterial action (silver) [4–9]. However, concerns have been raised with respect to their biological effect after unintended exposure [10–14].

Noble metal nanoparticles such as Au, Pt, Pd, and Rh have distinct catalytic properties in biology, and have been reported to show several enzyme-like activities in vitro, including reactive oxygen species scavenger activity [15–22]. Some authors have reported antimicrobial activity of gold, platinum, and palladium nanoparticles in the size range of 5 to 30 nm against gram-negative and gram-positive bacteria [23–25] and distinct adverse biological effects such as genotoxicity, induction of apoptosis and cell cycle arrest of human cells [24,26–27], whereas in other studies, these effects were not observed [28–30]. With ultrasmall gold nanoparticles and gold clusters, different biological events are triggered that can lead to a higher cytotoxicity of very small particles (<3 nm) [31]. Very little is known about the biological effects of rhodium nanoparticles except that these nanoparticles can penetrate into human skin [32]. Silver nanoparticles are applied in various fields including healthcare and biomedicine due to their antimicrobial, antifungal and antiviral effect [33–37]. This is based on the oxidative release of silver ions [38–43], which may affect biological systems in different ways, for example, by disruption of the cell wall or by interaction with cellular enzymes [44].

However, it is often difficult to compare literature reports because typically only nanoparticles of one metal are prepared and analysed. Furthermore, their surface functionalization, charge, and shape are usually different, so that a biological effect cannot be readily elucidated [45–47]. Thus, the nanoparticles cannot be compared with respect to their biological action [11–13,48–52]. This previously prompted us to carry out a multicentre study where the same silver nanoparticles were investigated in different laboratories with different biological assays [44].

Following the same approach, we have synthesized and thoroughly characterized nanoparticles of the noble metals Rh, Pd, Ag, Pt and Au that are of the same size and have the same surface functionalization (PVP) to compare their biological effect on a well-established system, i.e., on human mesenchymal stem cells (hMSC).

## Materials and Methods

### Chemicals

Poly(*N*-vinyl pyrrolidone) (PVP K 30, Povidon 30; Fluka, *M* = 40,000 g mol^−1^), sodium borohydride (Sigma-Aldrich, ≥96%, p.a.) trisodium citrate (Acros, anhydrous 98%), tannic acid (Fluka, p.a.), and D-glucose (Baker, p.a.) were all used as obtained. Aqueous solutions of H_2_PtCl_6_ or HAuCl_4_ (prepared by dissolution of platinum or gold in aqua regia), RhCl_3_, NaPdCl_4_, (both from Sigma-Aldrich), and AgNO_3_ (Roth, >99.9% p.a.), were used as metal precursor compounds. Ultrapure water (Purelab ultra instrument from ELGA) was used in all experiments. Before use, all glassware was cleaned with boiling aqua regia.

### Nanoparticle synthesis

**PVP-stabilized Rh, Pd, and Pt nanoparticles:** In a 100 mL two-neck round-bottom flask, 600 mg D-glucose and 50 mg PVP were dissolved in 50 mL water under stirring and heated to 100 °C under reflux. Then an aqueous solution (5 mL) of the corresponding metal precursor (25 µmol for Pt and 50 µmol for Rh and Pd) was rapidly added. The mixture was boiled under reflux for 60 min (Pd), 120 min (Rh), and 240 min (Pt), respectively. At the end of reaction time, the mixture was rapidly cooled to room temperature in an ice bath. Most of the water and the synthesis by-products were removed by centrifugation (4,000 rpm) in a spin filter tube (Millipore; molecular weight cut-off 3 kDa). The nanoparticles were purified by a final centrifugation step at 66,000 g for 30 min, followed by redispersion in ultrapure, degassed water. The dispersion was stored under argon at 4 °C to avoid oxidation. The yield was about 65 to 85% with respect to the metals as determined by atomic absorption spectroscopy (AAS).

**PVP-stabilized Ag nanoparticles:** In a 50 mL two-neck round-bottom flask, 1.80 mg AgNO_3_ and 1.80 mg trisodium citrate were dissolved in 35 mL water. The mixture was rapidly cooled in an ice bath with stirring (700 rpm). Then 1 mL of a 10 mM NaBH_4_ solution (dissolved in cold water) was added rapidly to the reaction mixture. After 1 min, an aqueous solution of 25 mM PVP (1 mL) was added and kept stirring for 3 h. The PVP-stabilized Ag nanoparticles were purified by double centrifugation (29,400 g) and redispersion in ultrapure, degassed water. The dispersion was stored under argon at 4 °C to avoid oxidation. The yield was about 25% with respect to silver as determined by AAS.

**PVP-stabilized Au nanoparticles:** In a 250 mL two-neck round-bottom flask, 600 mg trisodium citrate and 50 mg tannic acid were dissolved in 150 mL water under stirring and heated to 100 °C under reflux. Then, 5 mL of an aqueous HAuCl_4_ solution (containing 25 µmol Au) was added rapidly to the boiling solution. After 5 min, the reaction mixture was rapidly cooled to room temperature in an ice bath. 50 mg of PVP dissolved in 5 mL H_2_O was added for stabilization. The mixture was stirred for at least 12 h. Most of the water and the by-products were removed by centrifugation (4,000 rpm) in a spin filter tube. The nanoparticles were purified by a final centrifugation step at 66,000 g for 30 min and redispersed in ultrapure degassed water. The dispersion was stored under argon at 4 °C to avoid oxidation. The yield was about 70 to 80% with respect to gold as determined by AAS.

All synthesis parameters are compiled in Table 1.

**Table 1:** Synthesis parameters.

Metal	Metal / µmol	Reducing agent	Reducing agent / mg	Reaction time / min

Rhodium	50	D-glucose	600	120
Palladium	50	D-glucose	600	60
Silver	6	NaBH_4_	1.8	180
Platinum	25	D-glucose	600	240
Gold	25	citrate/tannic acid	600/50	5

### Characterization

All metal concentrations were determined by atomic absorption spectroscopy (AAS) with a Thermo Electron M-Series spectrometer (graphite tube furnace according to DIN EN ISO/IEC 17025:2005) after dissolving the particles in aqua regia (Rh, Pd, Pt, Au) and nitric acid (Ag), respectively.

Analytical disc centrifugation (differential centrifugal sedimentation; DCS) was performed with a CPS Instruments DC 24000 disc centrifuge (24,000 rpm). Two sucrose solutions (8 wt % and 24 wt %) formed a density gradient which was capped with 0.5 mL dodecane as a stabilizing agent. The calibration standard was a poly(vinyl chloride) (PVC) latex in water with a particle size of 483 nm provided by CPS Instruments. The calibration was carried out prior to each run. A sample volume of 100 μL was used.

Dynamic light scattering (DLS) for particle size analysis and zeta potential determination was carried out on a Malvern Zetasizer Nano ZS ZEN 3600 instrument (25 °C, laser wavelength 633 nm). The scattering was monitored at a fixed angle of 173° in backward scattering mode. The primary data were derived from the correlation function of the scattered intensity as a number-weighed size distribution.

Ultraviolet–visible (UV–vis) spectroscopy was performed with a Varian Cary 300 instrument from 200 to 800 nm with background correction. Suprasil^®^ cuvettes with a sample volume of 750 µL were used.

High-resolution imaging was performed using an aberration-corrected FEI Titan transmission electron microscope (TEM) equipped with a Cs-probe corrector (CEOS Company) and operating at 300 kV [53].

### Cell biology

Human mesenchymal stem cells (hMSC, 5th to 10th passage, Lonza, Walkersville Inc., MD, USA) were cultured in cell culture medium RPMI1640 (GIBCO, Invitrogen GmbH, Karlsruhe, Germany) containing 10% fetal calf serum (FCS, GIBCO, Invitrogen GmbH) and L-glutamine (0.3 g L^−1^, GIBCO, Invitrogen GmbH) using 75 cm^2^ culture flasks (Falcon, Becton Dickinson GmbH, Heidelberg, Germany). The cells were maintained at 37 °C in a humidified 5% CO_2_ atmosphere and sub-cultivated every 7–14 days, depending on the cell proliferation. Adherent cells were washed with phosphate-buffered saline solution (PBS, GIBCO, Invitrogen GmbH) and detached from the culture flasks by the addition of 0.2 mL cm^−2^ 0.25% trypsin/0.05% ethylenediaminetetraacetic acid (EDTA, Sigma-Aldrich, Taufkirchen, Germany) for 5 min at 37 °C. Subsequently, the hMSC cells were collected and washed twice with RPMI1640/10% FCS.

Subconfluent hMSC cells were seeded in 24-well cell culture plates (Falcon, Becton Dickinson GmbH, Heidelberg, Germany) at a density of 1.5 × 10^4^ cells per well and incubated for 24 h at 37 °C under cell culture conditions. Nanoparticle solutions of 1.0, 0.5, 0.2, 0.1 and 0.05 g L^−1^ concentration were prepared in sterile, ultrapure water by serial dilution. To each solution, 50 µL was added per mL of sample to achieve the final metal concentrations of 50, 25, 10, 5.0 and 2.5 mg L^−1^ (ppm) Adherent hMSC cells were then incubated in the presence or absence of different nanoparticle concentrations for 24 h in RPMI1640/10% FCS at 37 °C and 5% CO_2_. The cell viability and morphology of the incubated cells were analysed with calcein acetoxymethyl ester (calcein-AM, Calbiochem, Schwalbach, Germany) fluorescence staining. For this, the cells were incubated with 1 μM calcein-AM for 30 min at 37 °C under cell culture conditions and subsequently analysed by fluorescence microscopy (Olympus MVX10, Olympus, Hamburg, Germany). The quantification of cell viability was performed by phase analysis (CellSens Dimensions, Olympus, Hamburg, Germany) of calcein-positive fluorescence signals calculating the fluorescent area.

For calculating the phase analysis data, a threshold was set which clearly discriminated the calcein-positive cells from background. The calculated fluorescent area of nanoparticle treated hMSC was given as percentage of the nontreated control area, which was set at 100%.

## Results and Discussion

A number of physical and chemical methods to synthesize nanoparticles of noble metals has been described in the literature [54]. The shape-controlled synthesis of noble metal nanostructures can be achieved by different experimental procedures that aim to control nucleation, crystal growth, and finally, colloidal stabilization [55–58]. A very prominent method is the polyol process where compounds like ethylene glycol act as the solvent, reducing, complexing and stabilizing agent at the same time [59]. In general, chemical methods are usually based on the reduction of dissolved cationic metal species by suitable reducing agents [1]. Physical methods like laser ablation have also gained increasing importance in the last decade [60].

Rhodium nanoparticles can be prepared by reduction in the presence of suitable capping agents. The manipulation of the reaction kinetics by variation of synthesis parameters such as temperature and concentration leads to nanoparticles of different size and shape [61–62]. A hydrogen-based reduction in alcohols like methanol, heptanol, and propanol leads to sponge-like rhodium nanostructures [63]. Palladium nanoparticles can be synthesized either by the polyol process or by sono-, electro- and wet-chemical methods [64]. The reduction with sodium borohydride leads to small palladium nanoparticles (<10 nm) [65].

Silver nanoparticles can be synthesized in many different ways [66–67]. A bottom-up synthesis of silver nanoparticles can be performed either in organic solvents like ethylene glycol (EG), oleylamin (*cis*-1-amino-9-octadecene, OAm) [68], or in aqueous solutions. Depending on the additives and the reaction conditions, a variety of different shapes and sizes can be realized [66–67,69–74]. The generation of small silver nanoparticles (3–6 nm) is often performed only to generate seeds as a precursor for subsequent more complex morphologies, for example, as the core for bimetallic nanoparticles or as a sacrificial core for hollow nanoparticles of a more noble metal like gold [71,75–78]. There are many examples of syntheses for silver nanoparticles with a diameter between 5–10 nm [71,79–80]. Platinum nanoparticles can be obtained by metal carbonyl-mediated synthesis in organic solvents [81]. Nanoscale platinum catalysts were synthesized using NaBH_4_ in octylamine [82].

Small gold nanoparticles (<10 nm) can be produced by wet-chemical approaches using strong reductants (e.g., NaBH_4_) in the presence of strong-binding capping agents, but they can also be synthesized by the addition of tannic acid during the classic Turkevich method where citrate is both the reducing and the stabilizing agent [83–84].

In the following, we describe how we have prepared nanoparticles of noble metals with a diameter between 4 and 8 nm by different syntheses that are colloidally stabilized by PVP. Rhodium, palladium, and platinum were reduced with glucose, silver was reduced with NaBH_4_, and gold was reduced with a mixture of citrate and tannine. The particles were characterized by dynamic light scattering (DLS), analytical disc centrifugation (differential centrifugal sedimentation, DCS), ultraviolet (UV) spectroscopy, and high-resolution transmission electron microscopy (HRTEM). All characterization data are summarized in Table 2.

**Table 2:** Size distribution data of PVP-stabilized noble metal nanoparticles (all nanoparticles were well dispersed in water and not agglomerated). The polydispersity index (PDI from DLS) was between 0.1 and 0.3 in all cases, indicating a good monodispersity.

Metal	*d*(DLS) / nm^a^	*d*(DCS) / nm	*d*(TEM) / nm	Zeta potential / mV	Reducing agent

Rhodium	6.2 ± 1.7	4.1 ± 1.4	4.3 ± 1.2	+3 ± 3	Glucose
Palladium	10.0 ± 1.6	5.6 ± 1.9	4.6 ± 1.2	−3 ± 7	Glucose
Silver	10.1 ± 1.5	9.5 ± 1.3	8.2 ± 2.0	−27 ± 9	NaBH_4_
Platinum	8.5 ± 1.6	5.5 ± 1.4	5.0 ± 1.3	−13 ± 8	Glucose
Gold	5.6 ± 1.7	7.2 ± 1.2	5.8 ± 1.3	−9 ± 8	Citrate/tannic acid

^a^Size distribution by number.

**Figure 1:**
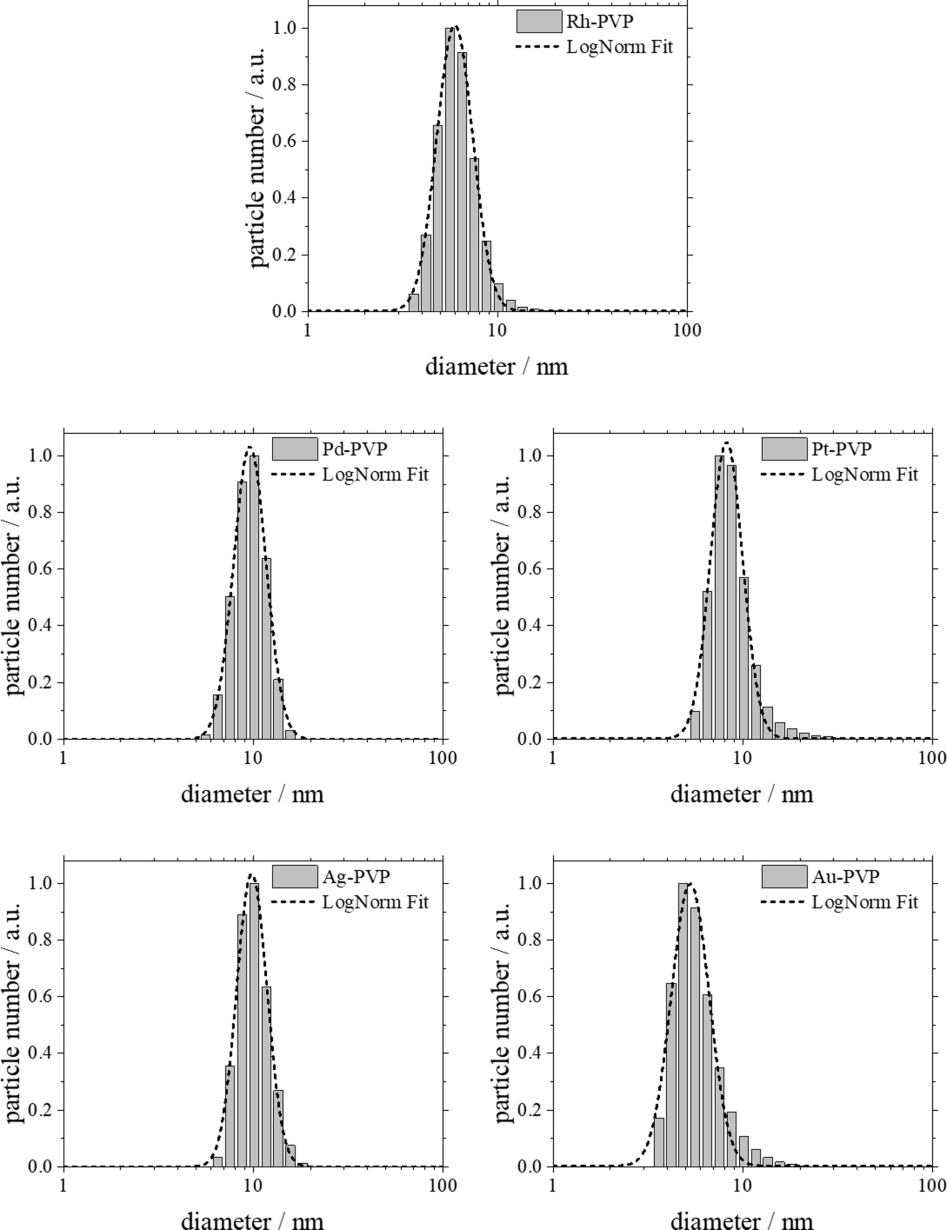
Particle size distribution of PVP-stabilized noble metal nanoparticles determined by dynamic light scattering (DLS). All data are given as size by number and are normalized for better comparison. The polydispersity index (PDI) was between 0.1 and 0.3 in all cases.

**Figure 2:**
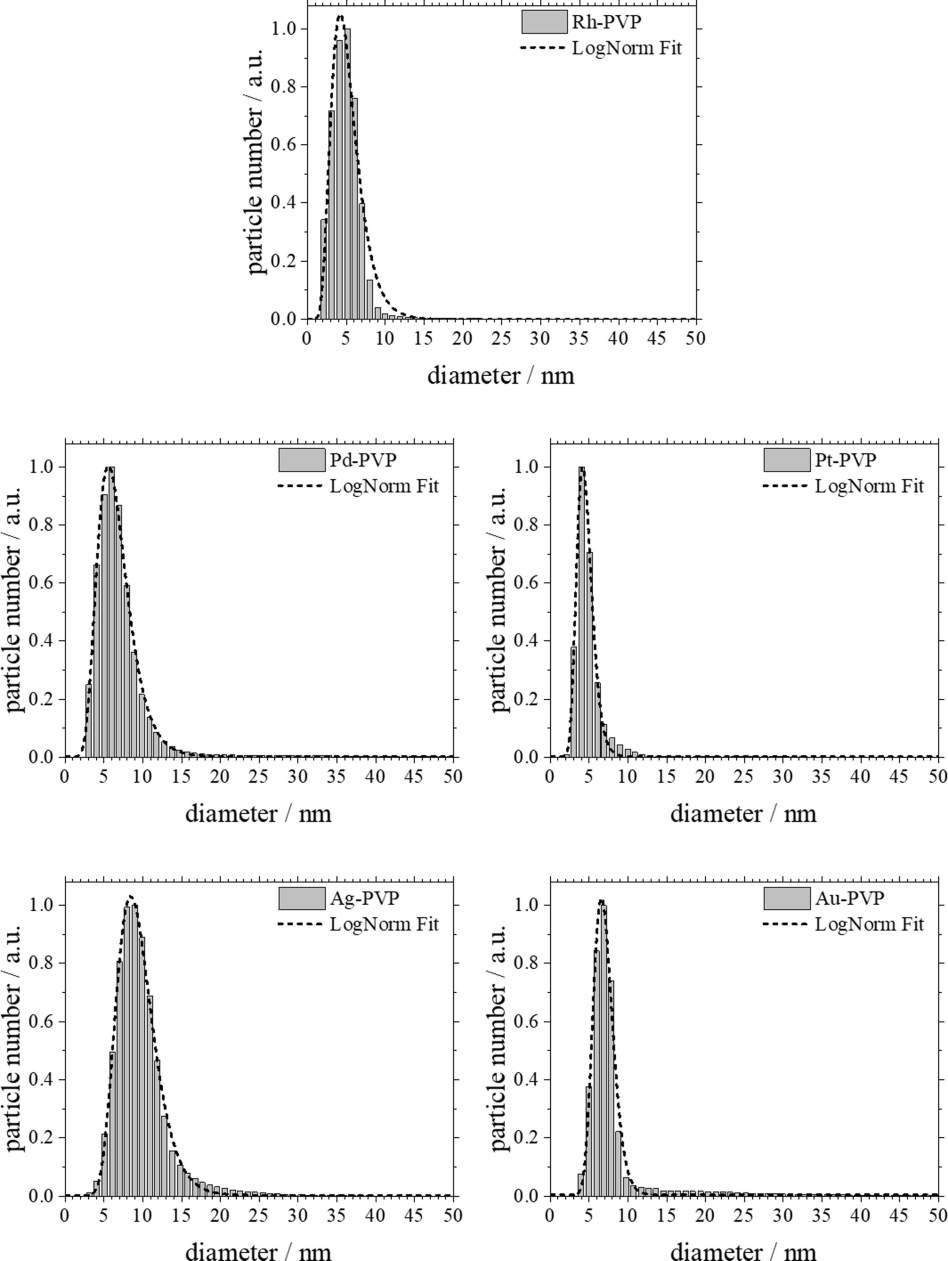
Particle size distributions of noble metal nanoparticles determined by analytical disc centrifugation (disc centrifugal sedimentation; DCS). All data are given as size by number and are normalized for better comparison.

**Figure 3:**
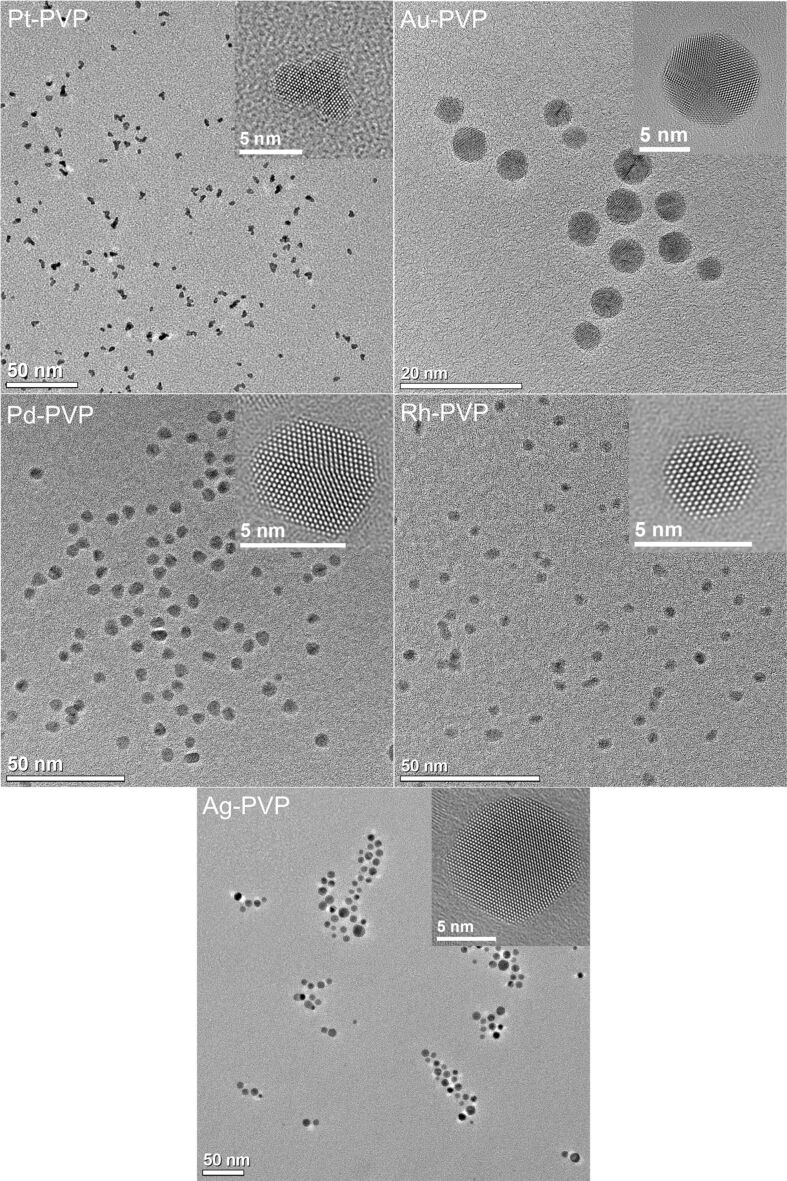
Representative high-resolution TEM images of PVP-stabilized noble metal nanoparticles (Rh, Pd, Pt, Ag, Au). In each upper right corner, a typical nanoparticle is shown in higher magnification.

**Figure 4:**
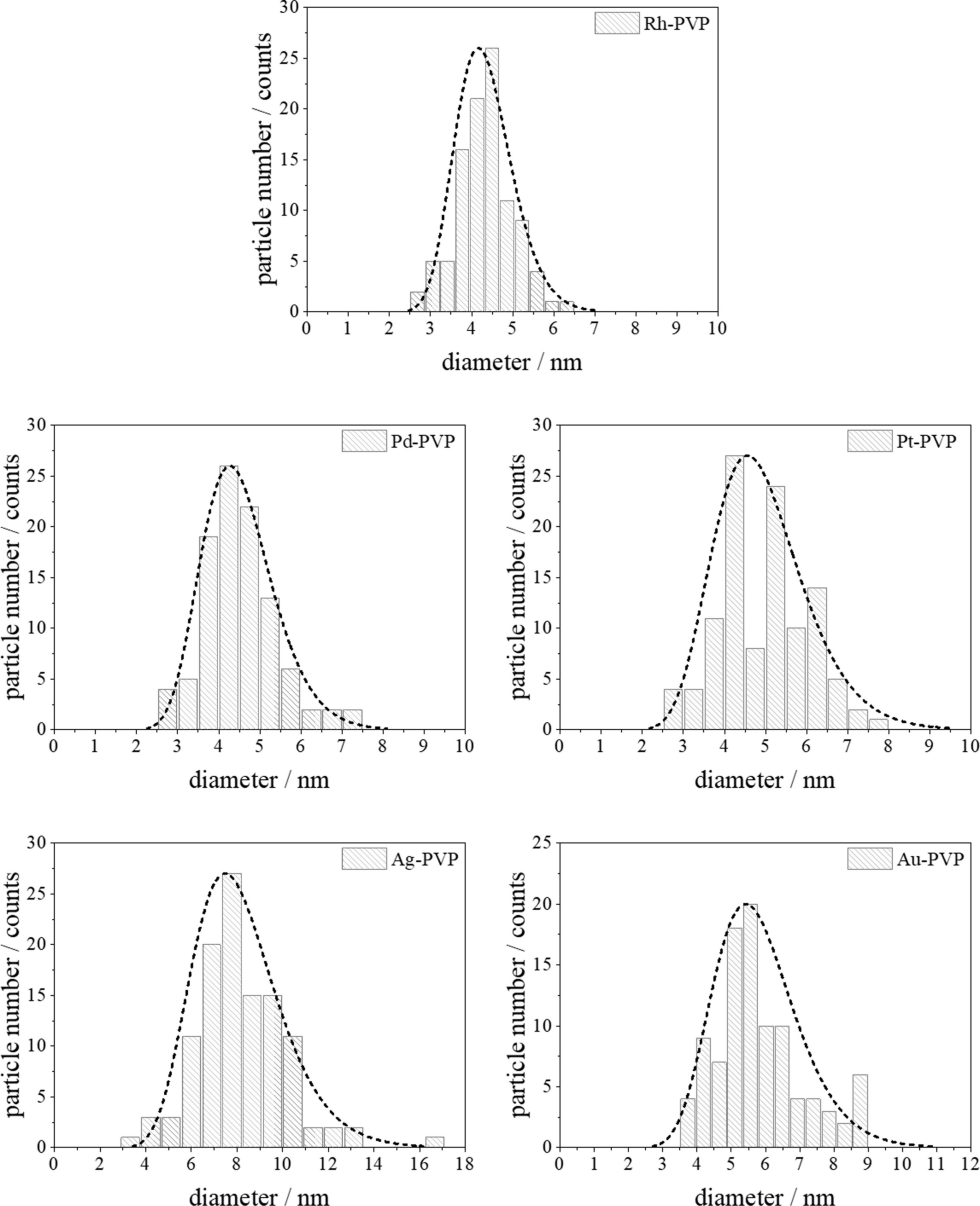
Particle size distributions of noble metal nanoparticles determined by high-resolution TEM imaging (HRTEM; log-normal particle size distribution fit). The histograms were analysed using a log-normal distribution fit to determine the number-weighted diameter.

All nanoparticles have a neutral or negative zeta potential. This is probably due to synthesis by-products that were not completely replaced by the stabilizing agent PVP [85]. Figure 1 and Figure 2 show the particle size distribution data from DLS and DCS, respectively. The average hydrodynamic diameter of the water-dispersed nanoparticles represents the diameter of the solid metallic core together with the surrounding hydrated PVP layer. The fact that the hydrodynamic diameter from DCS and from DLS is comparable with the size of the metallic core as determined by TEM (Table 1; Figure 3 and Figure 4) confirms the well-dispersed state of the nanoparticles in water without a significant degree of agglomeration – a prerequisite for meaningful cell culture studies [47,86]. Note that the hydrodynamic diameter determined by DCS is systematically underestimated because of the hydration shell [87].

The average diameter of all particles was in the range between 5 and 10 nm. These results agree well with the results from analytical disc centrifugation (Figure 2) and with the results from high-resolution transmission electron microscopy (Figure 3 and Figure 4).

High-resolution electron microscopy showed nanoparticles of almost spherical shape with diameters that are in good agreement with the results from DLS and DCS (Figure 3). The branched structures in the case of platinum indicate an aggregation of smaller particles during the synthesis. Whereas gold shows multiple twinning of crystals, silver appears to be single crystalline with only a few intrinsic stacking faults.

The specific properties of nanomaterials originate from the large surface-to-volume ratio and the local configuration of atoms [69]. The morphology of nanoparticles is defined by the contributions of the cohesive energy, the surface energy, the twinning energy, and the strain energy [88]. Based on the Wulff construction, the equilibrium shape of a nanocrystal can be predicted by minimization of the surface free energy of the crystal for a given enclosed volume. According to the Wulff construction, face-centered cubic (fcc) crystals tend to form truncated octahedral particles with large {111} and small {100} surfaces [89]. It was shown by Ino that metal nanoparticles (Ag, Au, Cu, Pd, Pt) prefer an icosahedral structure if their diameter is smaller than approximately 10 nm. Nanocrystals with larger diameters prefer the shape of a truncated octahedron [88,90].

**Figure 5:**
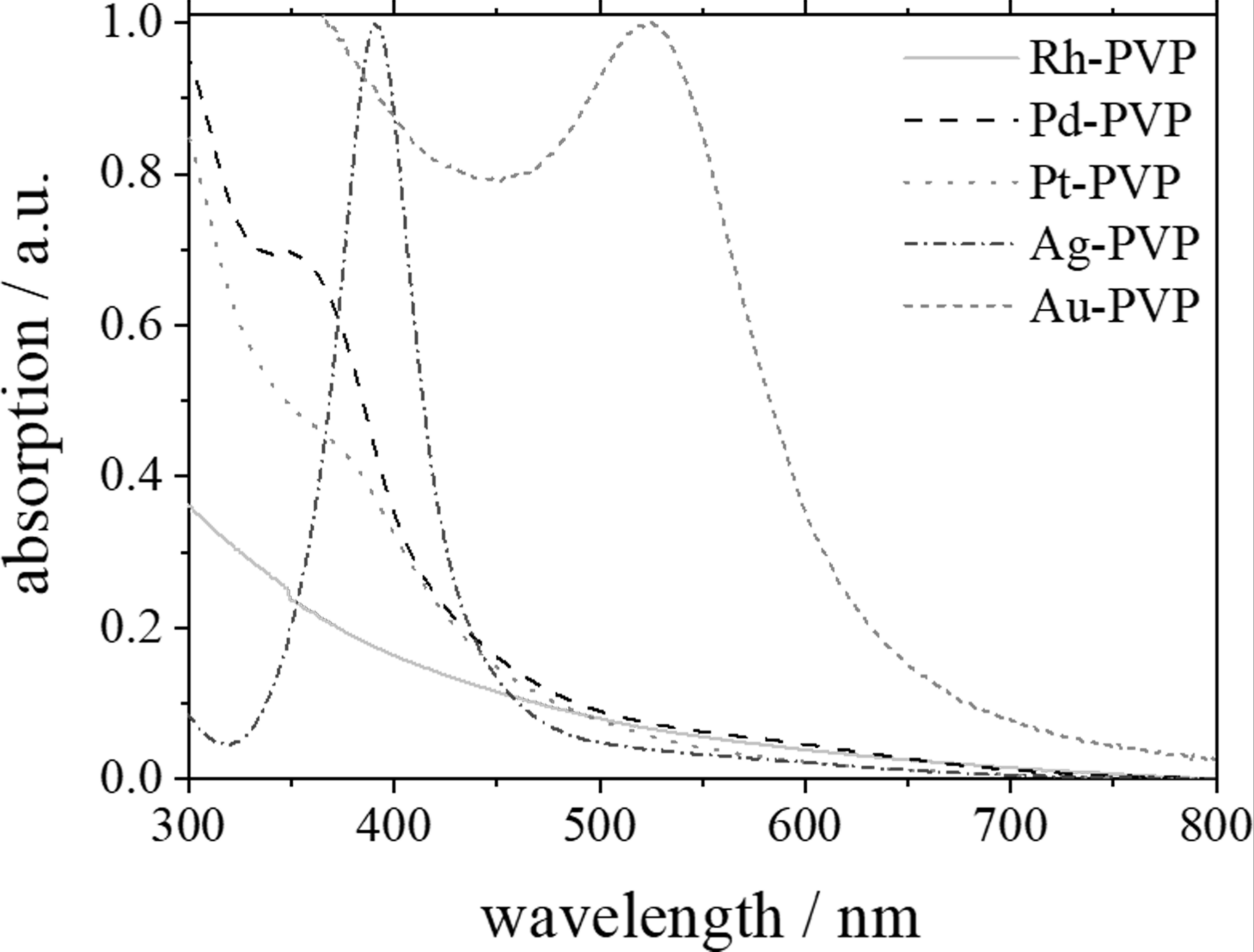
UV–vis absorption spectra of PVP-stabilized noble metal nanoparticles (Rh, Pd, Pt, Ag, Au).

UV–vis spectroscopy gives information on the optical properties of nanoparticles, e.g., surface plasmon resonance (SPR) effects (Figure 5). While the dispersions of platinum group nanoparticles (Rh, Pd, Pt) were all brown-black and had no distinct absorption in the visible range, silver and gold showed the typical surface plasmon resonance (SPR) absorption bands [91].

**Figure 6:**
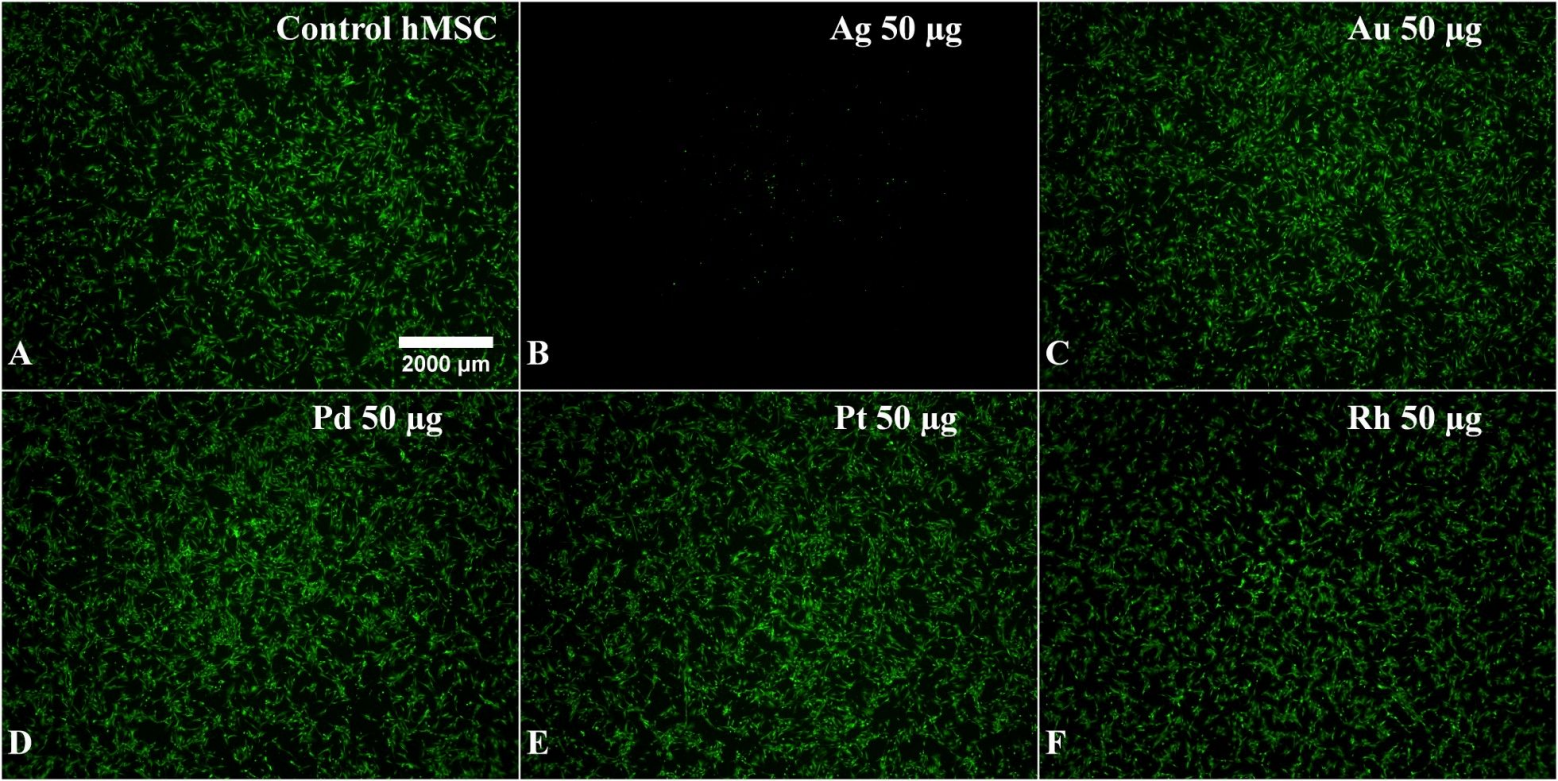
Effects of nanoparticles on the viability and the morphology of human mesenchymal stem cells (hMSCs). hMSCs in 24-well cell culture plates were incubated with different nanoparticles (50 µg mL^−1^ = 50 ppm) under cell culture conditions and subsequently stained with calcein-AM. Viable cells are indicated by green fluorescence (A: control: untreated hMSC).

**Figure 7:**
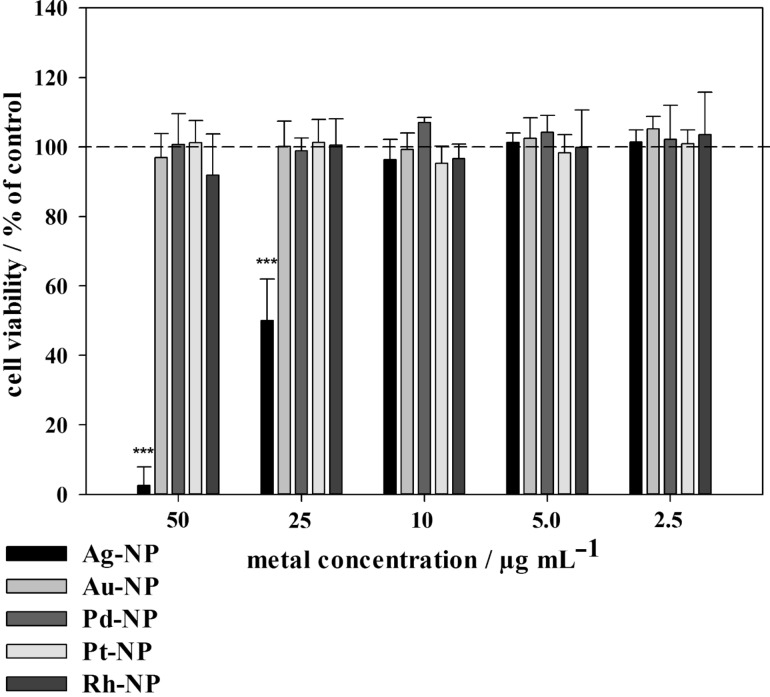
Influence of noble metal nanoparticles on the viability of human mesenchymal stem cells. The cells were treated with 2.5 to 50 µg mL^−1^ for 24 h under cell culture conditions. Viable cells were quantified by digital image processing (phase analysis). The data are expressed as mean ± SD (*n* = 5 independent experiments) given as the percentage of the control (cells cultured without nanoparticles). Asterisks (*) indicate significant differences in comparison to the control (***p ≤ 0.001).

In order to analyse the influence of noble metal nanoparticles on the viability of cells, human mesenchymal stem cells (hMSCs) from bone marrow were cultured in the presence of different nanoparticles (50 µg mL^−1^) under cell culture conditions. All nanoparticles were easily dispersible in cell culture medium and no agglomeration or sedimentation was observed. Only silver nanoparticles had a discernible effect on the viability of hMSC after 24 h of exposure (Figure 6). They had a cytotoxic effect starting at 25 µg mL^−1^. The cell viability further decreased with increasing silver concentration from 25 to 50 µg mL^−1^ (Figure 7). These effects are well known for silver, silver chloride and silver ions [33,38,44,92]. Cell detachment was only observed at toxic concentrations of silver nanoparticles (25 to 50 µg mL^−1^), but not at subtoxic concentrations (2.5 to 10 µg mL^−1^). Thus, cell detachment is correlated to cell toxicity. It has been reported that silver nanoparticles adversely affect the differentiation of hMSC at subtoxic concentrations [93].

In contrast, no influence of gold, palladium, platinum, or rhodium nanoparticles on the morphology of adherent hMSC was observed, even at higher metal concentrations (50 µg mL^−1^). Even at prolonged incubation times (up to 7 days), no cell toxicity was observed (data not shown) for these metals. Neither agglomeration nor sedimentation of the nanoparticles was observed in the cell culture medium in any case, indicating good dispersion. As the toxicity of silver nanoparticles is due to the oxidative release of silver ions [38–40,43,67,94–100], we can tentatively assume that such a dissolution does not occur for the more noble metals, and that the nanoparticles themselves are not cytotoxic. Due to the more noble character of gold, palladium, platinum, or rhodium in comparison to silver, oxidation by dissolved oxygen is not expected from an electrochemical point of view.

## Conclusion

Spherical nanoparticles of noble metals (Rh, Pd, Ag, Pt, Au) with an average diameter between 4 and 8 nm were prepared in aqueous media using different reducing agents. In addition, an accurate characterisation was performed, indicating a high degree of nanoparticle monodispersity and synthesis reproducibility. Except for silver, there was no adverse effect on human mesenchymal stem cells (hMSCs) up to a concentration of 50 ppm. In particular, no negative effect was observed for rhodium, palladium, platinum, and gold nanoparticles up to 50 ppm, indicating that no oxidative release of ions occurs. We conclude that there is no adverse effect of nanoparticles of these very noble metals on the cells because they are chemically inert and do not release toxic ions.
